# Electricity and “Painless Dentistry”

**Published:** 1894-08

**Authors:** L. E. Custer

**Affiliations:** Dayton, Ohio


### Electricity and "Painless Dentistry."

BY L. E. CUSTER, B.S., D.D.S., DAYTON, OHIO.

Read before the 38th, Annual Meeting of the Michigan Dental Association.

No one has told us what it is, yet it is an agent in the formation, growth and death of all animal and vegetable organisms; it came with the light and the heat giving the breath of life and the magic of colors. It is the unseen hand at work in nature, the cause of barometric changes, and the formidable agent which now and then shatters and tears the atmosphere, terrifying you with the crash of its thunder. It is that which transmits the enchantment of a look and the grace of a smile. It lights and heats our homes, it runs our factories and cars, and will soon carry our voice around the globe. In this decade when everything fairly sparkles with electrical phenomena it is not surprising that an agent which exhibits such a variety of wonders and which promises still more, should find a place in dental practice. At the same time, in the present state of the public mind, no agent can be more delusive in the hands of a fairly-skilled dental charlatan than electricity; yet when intelligently used by the careful operator, even in its present limited application, it goes far toward mitigating the pain of many operations. There is perhaps no broader field for the application of the various forms of electricity than in dental practice.

The similarity between the transmission of nerve force along a nerve fiber and electricity along a conductor is so marked that it is quite natural, at first thought, to suppose it possible to apply an electric current to a nerve force in such a manner and strength as to stop the neural activity and thus prevent the transmission of sensation. Indeed, DuBois-Raymond says: "If the galvanic current moves through the nerve in the same direction as the nerve current, the intensity of the latter is increased and the nerve current is diminished in intensity by the passage of the galvanic current in the opposite direction." But the effective application of electricity to the above end as a specific obtundent is so uncertain that with our present knowledge it is not practical. This statement may be a disappointment to some, but it is nevertheless true.

The profession has experimented with electrical anæsthesia since 1851. At first it created such a furore as to be called the "lightning anæsthetic." Many dentists and surgeons engaged in its use, but it was so erratic in its action that it was finally thrown aside by the more intelligent practitioners. It has been used more or less ever since, however, by the charlatans and that class of people who maintain that there *must* be some virtue in so wonderful an agent. They have given it new names, they have put it in new boxes; but whether it is the "electric vibrator" with musical accompaniment, it is the same old foadic current of forty years ago. Its application is painful. According as the current is increased the actual pain is overcome by a greater, the electrical pain. It is a matter of choice with the patient whether he will have substituted an electrical pain for the real. The substitution of one kind of pain for another is not professional. The shocking machine which does this is of no real value. Our object is to lessen the pain. When we can successfully balance neural activity by an application of electricity in such a manner as to be painless, as well as effective and practical, then we can speak of such a thing as electrical anæsthesia.

It is not absolutely necessary that electricity be an obtundent itself for admission into dental practice. There are other ways in which it can be advantageously used for reducing the pain of many dental operations, and in this connection I will state that reference to a few electrical instruments of my own device is not for any commercial purpose. These instruments have all been made to meet some need in my own practice and are here presented as illustrations of the subject at hand.

If, with the aid of electricity, we can shorten an operation without increasing the pain, we have done a good thing, or if in the same amount of time we can perform a better operation, we are progressive. The constant potential of one hundred and ten volts is the ideal current for dental purposes. It is perfectly safe. It furnishes an abundance of power for the engine and heat for the heaviest cautery. It gives the best of light, and is admirably adapted for electrolytic purposes.

The perfection of electric motive power for the dental engine can only be fully appreciated by one who has used it. The demand has been made for a sharp bur revolved at a high rate of speed for the painless removal of sensitive dentine. This is perfectly met by the electric motor. It not only gives a much higher rate of speed than is obtainable by other power but the variations of speed by means of the rheostat is under the perfect control of the operator. The bur may be instantly stopped or its direction reversed so that there is little damage to the tooth, gum or rubber cloth by accidental movement of the instrument.

A most important recommendation also of electric motive power is that the body of the operator may be perfectly quiet, so that the control of the hand-piece is much more delicate than where the treadle engine is used.

Electric heat depends upon two factors—the quantity of the current and the size and the conductive power of the conductor. It differs in some respects from heat produced by the combustion of gas or alcohol and for certain purposes these new qualities make it a valuable agent in dental practice. It may seem that the simple process of annealing gold does not bear upon "painless dentistry," but if the contour of a large gold filling has dropped off by the imperfect cohesion of a smoked, gased or poorly-annealed piece of gold, necessitating a second operation with all the pain and fatigue that such cases mean, not to speak of the mortification to the dentist, I would say it has a most direct bearing. The heat produced by electrically heating a mat of coiled platinum wire is the cleanest, most uniform and most accurately controlled of all forms of heat. The cohesive property of pure gold is supposed to be developed by heating to such a temperature as to drive off the gases condensed upon its surface, principal of which is ammonia. The alcohol and Bunsen flame are ordinarily used for this purpose, but who is certain but that better results may not be obtained by subjecting to a heat free from the products of combustion as well as to the danger of smoking and the exposure to unconsumed gases? The electric annealer effectually overcomes these dangers. The heat being derived from electrically-heated platinum, one of the noble metals, is absolutely free from gas of any kind. The heat derived from a mat of platinum coils is quite uniform at all parts so that the gold is not only thoroughly annealed but to the same degree. It is impossible to evenly heat a piece of gold held with a pair of pliers over a flame of any kind. The thin edges of the gold will be fused while the part between the pliers will be scarcely warm. "Batting" of gold is due almost entirely to unequal annealing. At a clinic not long ago I witnessed an operator annealing gold pellets with a pair of foil carriers the points of which were broad as the pellet itself. The result was that the exposed edges of the pellet were a fused mass while the part between the plier points was not even made to cohere. The result was that the unannealed portion was not cohesive and the annealed portion so stiff that it was with difficulty that enough surface was brought in actual contact to cohere. No wonder such operators lay the blame with the gold! The accuracy with which electrical heat can be controlled also recommends its use for annealing. By means of the rheostat any degree of heat to the melting point of platinum may be obtained. From my experience with the electric annealer, however, I find that cohesion is developed at a much lower degree than at first supposed. It is never necessary to heat even to redness alone, fusion. The heat may be so low that the gold may be subjected to it for hours or for days even without any injury and still be highly cohesive. If it were not my own invention, I would say next in usefulness to the dental engine is the electric gold annealer.

The electric cautery was a marked improvement over the actual cautery of years ago. A similar instrument may be used as a root dryer. The heat is not only steadily maintained but is under the absolute control of the operator. Instead of approaching the patient with the formidable red-hot iron, the instrument is placed in position, the button touched and the heat maintained or varied at will. By a shunt arrangement, the incandescent current may be used for the cautery and root dryer without injury to the instrument.

Every operator knows the difficulty of throwing a blast from the warm-air syringe without producing pain. It is not sufficiently definite. This difficulty may be overcome by the use of the electric current. Through a coil of German silver wire of a given size; a current at one hundred and ten volts gives heat which may be accurately measured. If this wire is enclosed in a rubber tube and through the tube air is forced at a given pressure it will escape heated to the desired degree. The temperature may be varied at the will of the operator by varying the heat of the coiled wire by a rheostat or by varying the air pressure. The calculations being accurate, by this device it is possible at all times to obtain a blast of air at blood heat which will not be painful.

The electric mallet is a time-saving appliance familiar to all. In many cases the peculiar blow from this instrument is more agreeable than any other form of malleting. The electric light is a valuable aid in diagnosis and many times saves drilling and other forms of exploration.

The appliances just mentioned are practical and useful instruments and are steps toward painless dentistry, but we have just begun the application of electricity in dental practice. I see in the near future an electro-deposition of gold or platinum in the cavity of a tooth which is not only a preventive of recurrent decay itself but is a cohesive foundation to which a gold filling may be attached relieving the walls of much strain. I also see a plating of metal covering the whole crowns of the posterior teeth predisposed to caries or in a less charlatan-like manner it may be restricted to the fissures and proximal positions.

